# Development of an Artificial Neural Network-Based Tool for Predicting Failures in Composite Laminate Structures

**DOI:** 10.3390/biomimetics10080520

**Published:** 2025-08-08

**Authors:** Milica Milic Jankovic, Jelena Svorcan, Ivana Atanasovska

**Affiliations:** 1 Faculty of Mechanical Engineering, University of Belgrade, 11120 Belgrade, Serbia; mmilic@mas.bg.ac.rs (M.M.J.); jsvorcan@mas.bg.ac.rs (J.S.); 2 Department of Mechanics, Mathematical Institute of the Serbian Academy of Sciences and Arts, 11000 Belgrade, Serbia

**Keywords:** failure prediction, artificial neural networks, composite materials, structural design, FEA

## Abstract

Composite materials are widely used in aerospace, automotive, biomedical, and renewable energy sectors due to their high strength-to-weight ratio and design flexibility. However, their anisotropic and layered nature makes structural analysis and failure prediction challenging. Traditional methods require solving complex interlaminar stress–strain equations, demanding significant computational resources. This paper presents a bio-inspired machine learning approach, based on human reasoning, to accelerate predictions and reduce dependence on computationally intensive Finite Element Analysis (FEA). An artificial neural network model was developed to rapidly estimate key parameters—laminate thickness, total weight, maximum stress, displacement, deformation, and failure criteria—based on stacking sequence and geometry for a desired load case. Although validated using a specific composite beam, the methodology demonstrates potential for broader use in rapid structural assessment, with prediction deviations under 15% compared to FEA results. The time savings are particularly significant—while conventional FEA can take several hours or even days, the ANN model delivers accurate predictions within seconds. The approach significantly reduces computational time while maintaining precision. Moreover, with further refinement, this logic-driven model could be effectively applied to aircraft maintenance, enabling faster decision-making and improved structural reliability assessment.

## 1. Introduction

Composite materials have become integral to industries demanding high structural performance, including aerospace, automotive, biomedical, and renewable energy sectors. Their superior strength-to-weight ratio and the flexibility to form complex geometries confer significant advantages. Typically composed of multilayered architectures with anisotropic properties, composites offer substantial benefits over conventional materials [1,2]. However, their complex mechanical behavior under various loading conditions presents considerable challenges in accurately predicting structural performance and failure mechanisms [3]. These challenges are further compounded by the necessity to account for a wide array of factors—microstructural features, material properties, geometric configurations, manufacturing processes, and environmental conditions—all of which must be carefully integrated into the design process [4]. The application of the finite element (FE) method is still predominant in the analysis and construction of aerospace structures [5].

In the early stages of engineering design, the rapid and reliable identification of mechanical properties under diverse service conditions is crucial for informed decision-making. Due to the combinatorial complexity of possible material architectures and operational environments, exhaustive experimental characterization becomes impractical. As such, machine learning (ML) approaches offer a promising alternative, enabling the identification of underlying relationships between material architecture and mechanical response under defined constraints [6,7,8,9,10,11]. These techniques facilitate the prediction of optimal stacking sequences to achieve target mechanical properties [12]. For laminated aerospace components, the prediction of failure is critical to the structural integrity and safety of aircraft [13]. In particular, interlaminar stresses that arise at layer interfaces are of paramount importance in understanding delamination and the initiation of failure. When these stresses exceed allowable limits, fiber fracture and delamination may occur, drastically compromising structural integrity [14]. The integration of fracture criteria into machine learning (ML) models—accounting for interlaminar stress states—enhances predictive accuracy, supporting the development of optimized structures capable of withstanding complex loading scenarios [15].

The implementation of artificial neural networks (ANNs) for failure prediction in composites provides significant economic and safety benefits. ANN-based models enable the early detection of failure-prone configurations, thereby reducing maintenance costs, extending service life, and improving system reliability. During the design phase, ANNs aid in material selection and reduce reliance on expensive and time-consuming experimental trials. Furthermore, with appropriate advancements, ANN models can be extended to support predictive maintenance strategies, enhancing operational reliability while minimizing inspection and testing costs.

This study proposes a machine learning-based methodology for failure prediction in composite structures, incorporating critical design variables such as ply orientation, laminate thickness, and in-plane stress components (σ_11_, σ_22_, σ_12_), grounded in established failure criteria. Only composite laminates made of a polymer matrix and fibers, with a predefined stacking sequence, are addressed. All layers are modeled as orthotropic and linearly elastic materials, without defects such as delamination, voids, or cracks. The methodology is applied to a UAV engine mount subjected to impact loading, with structural behavior characterized in previous investigations [16]. A comprehensive dataset is generated through parametric FE simulations, using input features including ply orientation, thickness, and hole diameter. Output parameters encompass mass, stress distribution, displacements, strain fields, and safety factors, derived from repeated structural evaluations.

Artificial neural networks, known for their high efficacy in modeling nonlinear relationships within complex datasets [17,18], are used to train the predictive model. Prior studies have demonstrated that ANNs are particularly well-suited for estimating tensile strength and failure in composite laminates [19]. The developed tool streamlines the design process by providing rapid feedback on laminate performance under variable loading conditions, enabling the accelerated identification of optimal configurations [20]. Moreover, integration with bio-inspired optimization algorithms facilitates efficient exploration of the design space, reducing computational overhead while maintaining prediction accuracy, validated through experimental results. This work presents a comprehensive framework that integrates FEA and machine learning for fracture prediction, further laminate optimization, as well as increased computational efficiency and reduced computational time. The developed tool serves as a valuable asset in the early phases of composite structure design, offering rapid and accurate predictions of material behavior and promoting the development of more reliable and efficient composite components, particularly in high-performance applications such as aerospace [21].

The authors acknowledge that the current version of the presented tool is tailored specifically for composite laminates and does not encompass the full spectrum of composite configurations, such as sandwich structures, or complex hybrid systems. This tool is primarily designed for fiber-reinforced laminates with a relatively straightforward stacking sequence and uniform material properties. Its applicability is currently limited to cases where laminate theory and classical failure criteria can be reasonably applied. Complex behaviors involving significant through-thickness effects, damage evolution, or multi-material interactions are beyond the intended scope at this stage.

## 2. Description and Validation of the Starting Structural Model

The configuration under consideration is a parametric model of a beam structure that incorporates a large number of geometric and structural input parameters. Geometric parameters include the beam dimensions, while structural parameters encompass material properties, meshing size, applied loads, and other pertinent factors. This model facilitates the analysis of various scenarios and produces output parameters, such as stresses, displacements, deformations, and safety factors, which serve as indicators of structural performance.

The beam has a circular cross-section with a diameter of 50 mm and a total length of 1670 mm. The weakened sections are located at two points on the beam, with the distance between the section axes being 1300 mm. The holes at the weakened sections are through-holes for mounting the electric motors. The holes on the bottom of the beam are 6 mm in diameter, while the ones on the top are 2 mm and contain threads. The weight of the six-layered laminate beam without the motor, with the vertical tail and half of the horizontal tail, is 3790 g, while the weight of only the part of the beam under consideration is 690 g.

**Figure 1:**
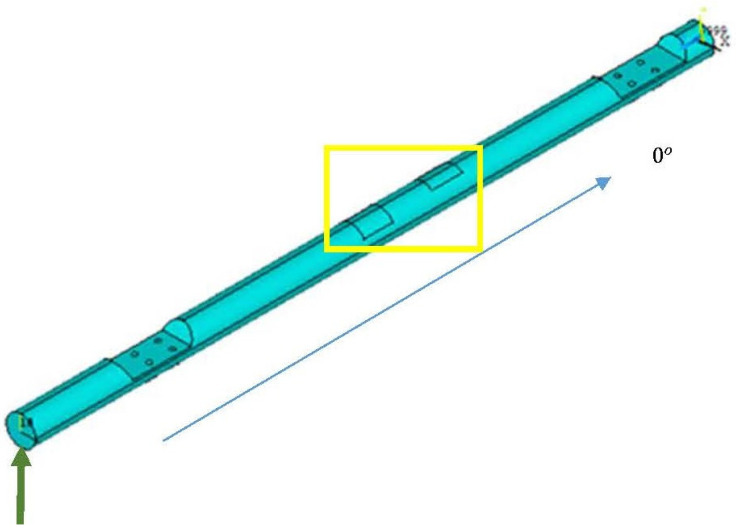
The developed parameterized model of the beam with illustrated boundary conditions (green arow represents external loading force, yellow square represents the constrained area, blue arow represents the main direction of the composite layers of the beam).

The load applied is a static resultant force F, which represents a force (F_avg_) in the vertical direction (*z*-axis), corresponding to thrust or impact force, and a force representing the tail aerodynamic effect (F_R2_) applied at a 30° angle to the vertical direction. The force is shown in Figure 1, as well as the parameterized model and the setup of FEA conditions. Contour conditions (constraints) are marked in yellow. The main direction of the layers is set at 0° relative to the longitudinal axis of the beam.

**Figure 2:**
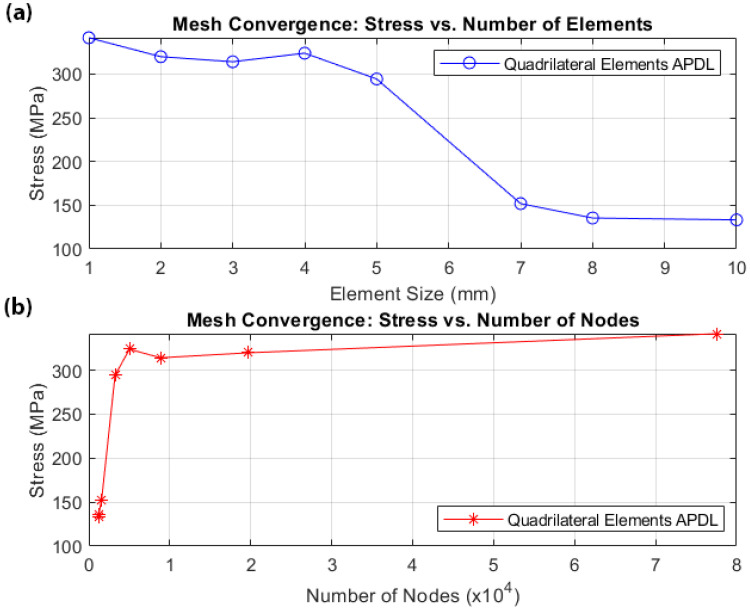
Mesh convergence study: (**a**) maximum stress versus element size; (**b**) maximum stress versus number of nodes.

The beam is discretized using rectangular six-layer shell elements, with the layering described by twelve variables (six ply thicknesses and six corresponding ply orientations that are also used in the predictive tool). Two additional input parameters representing hole diameters used for connection to other UAV elements are included in the study, resulting in a total of 14 input parameters. To ensure that the discretized finite element model does not significantly affect the results, a mesh convergence study was conducted. Several different element sizes, ranging from 10 mm to 1 mm, were tested under the same loading conditions. The values of the maximum stresses were compared with element size and node count, as illustrated in Figure 2a and 2b, respectively.

**Figure 3:**
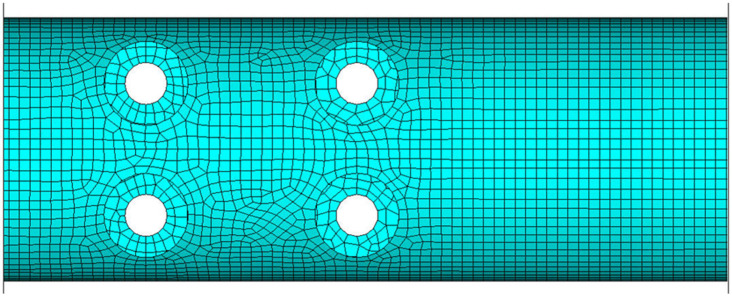
FEA mesh of the isolated beam segment.

Following the mesh convergence study, an element size of approximately 4 mm was adopted, resulting in a model with 10,975 nodes and 21,452 finite elements. A segment of this mesh is shown in Figure 3. The mechanical properties of the materials (corresponding to carbon plies) used in the beam FE simulations are provided in Table 1.

**Figure 4:**
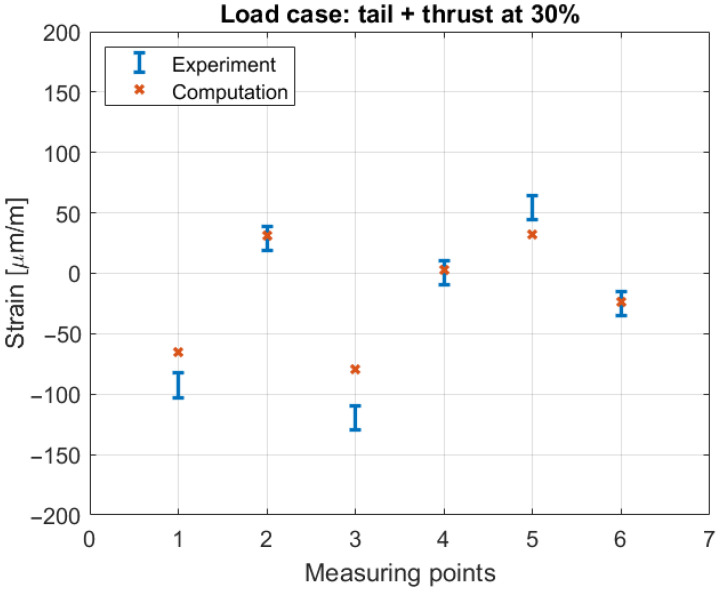
Comparison of computed and measured strain values.

To additionally validate the numerical model that forms the basis for the subsequent predictive tool based on ANNs, a simple static test was performed. Since all the details of the experimental setup and measuring equipment can be found in [22], here, just one load case will be presented. Figure 4 illustrates the correspondence between measured and computed strains at six locations distributed along the beam. It can be observed that the trend of the structural response to static loading is completely captured by the numerical model. Slight deviations in the absolute values can be attributed to the faults in the material, errors during the production process, and strain gauge sensitivity. The numerical model simulates ideal manufacturing conditions, which are never fully attainable in practice. Nevertheless, the observed trend of agreement between numerical and experimental results confirms the robustness and accuracy of the model, providing a reliable foundation for the development of a predictive tool based on artificial neural networks. Additionally, the model demonstrates the capability to accurately replicate the physical behavior of slender laminated structures under quasi-static loading.

## 3. Methodology

### 3.1. Methodology of Applying Machine Learning to Composite Materials

**Figure 5:**
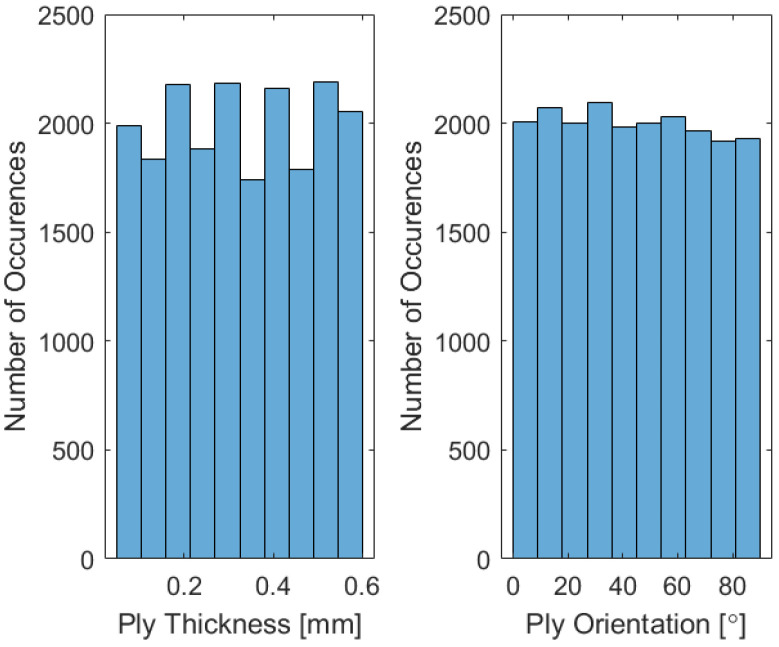
Nearly uniform distributions of the input parameters.

The input dataset is generated through a substantial number of structural simulations (20,000 repeated calculations) conducted on randomly generated configurations, representing potential solutions. Given that the starting structural model is based on the 14 mentioned independent input and 6 output parameters, this set size was determined as sufficient after trial and error. Here, the 14 input parameters, including ply thicknesses, ply orientations, and hole diameters, are nearly uniformly distributed within the design space to ensure sufficient data variability and generality (as can be seen in the histogram in Figure 5), but different choices can be made for different structures and materials.

Following the election of input parameters, a large number of repeated FEA simulations of the beam (as described in Section 2), under various loading conditions and with different layer stacking combinations, were used to form a database for the predictive model. This approach involves performing simulations across a broad spectrum of input parameters, including geometric characteristics, materials, loads, and boundary conditions, thereby ensuring representation of various operating conditions and their impact on structural behavior. The repeated FEA calculations enable the evaluation of the structure’s behavior across different parameters, facilitating the identification of critical points in the structure and a deeper understanding of the relationship between input and output data, such as stresses, displacements, deformations, total laminate thickness, mass of the entire structure, and safety factors. The use of these simulations provides quantitative data that is crucial for calibrating numerical models and validating predictive algorithms. Generating the database in this way not only allows for a detailed analysis of structural responses to various types of loads and changing operating conditions but can also serve as the foundation for the application of advanced optimization techniques and machine learning. Intending to increase accuracy and efficiency, such databases enable the development of robust models capable of delivering precise performance predictions under complex and uncertain conditions, thereby significantly enhancing the decision-making process in engineering design and analysis. In the existing literature, particularly in [23,24], four machine learning techniques—XGBoost, Random Forests, Gaussian Processes, and artificial neural networks (ANNs)—were applied to predict the tensile strength at the failure of the first and last laminate layers with various orientations (0°/90°/±45°). The findings demonstrated that artificial neural networks (ANNs) exhibited the best performance for larger datasets, making them the most suitable for designing composite structures. For this reason, artificial neural networks (ANNs) were selected for the development of the machine learning model methodology in this paper.

### 3.2. The Algorithm for Developing a Machine Learning Model for a Predictive Tool

The machine learning model applied to this class of engineering problems was developed as follows:

1. Geometry design

The development of the parametric geometry model under consideration involves creating a flexible and adaptable model that allows changes in the geometric characteristics of the structure through defined parameters. This approach enables fast and efficient design adaptation by varying key input parameters, such as dimensions, layer thickness, orientation, and other geometric factors, without the need for complete remodeling. The parametric geometry model is based on parameters that define the basic characteristics of the structure, enabling the automatic generation of new configurations and simulations in response to changes in these parameters. For example, beam dimensions, hole arrangements and dimensions, layer thicknesses, or layer angles can be represented as parameters that can be modified. This model enables changes to the geometry and facilitates the rapid testing of different boundary conditions, thereby improving the efficiency of the design process.

2. Database development

A database is created by pairing each set of input parameters (descriptors) with corresponding output parameters. The dataset is split into training, validation, and test subsets (70%, 15%, and 15%, respectively). A large number of structural simulations (20,000) were conducted, of which 18,000 were deemed valid for further analysis, while 2000 simulations were discarded due to geometric or structural inconsistencies.

3. Algorithm formation

Artificial neural networks (ANNs) are trained on the generated dataset to produce a model capable of predicting laminate thickness, mass, stress, displacement, strain, and failure criteria based on input parameters. The algorithm includes a fast evaluation of the desired results, the formation of a dataset, and the integration of a specific failure criterion, based on which the predictions are made. This algorithm enables the efficient and accurate prediction of outcomes, allowing the model to assess structural performance and identify failure points under various conditions:

- Rapid evaluation of individual solutions

The trained model allows the quick prediction of mass, displacement, maximum stress, and failure coefficient based on specified input parameters.

- Dataset

Each dataset entry pairs input parameters $x_{j}\left(q_{i}\right)$ with corresponding output parameters, represented in Equation (1).

(1) $$\left\{\left(x_{j}^{\left(1\right)},q_{i}^{\left(1\right)}),(\right.\right.\left.\left.x_{j}^{\left(2\right)},q_{i}^{\left(2\right)}),\ldots ,x_{j}^{\left(s\right)},q_{i}^{\left(s\right)}\right)\right\}$$

where j=1,…,n and i=1,…,m (n: number of input parameters (e.g., thickness, orientation, hole diameter); m: number of output parameters (e.g., stress, displacement, failure coefficient, mass, total thickness of laminate, strain)).

- Failure criteria (Tsai–Wu)

The core of this methodology is to adequately integrate the selected failure criterion, based on which the machine learning model predicts failure in the laminate or a specific structure. The Tsai–Wu failure criterion was introduced in 1970 by Stephen W. Tsai and Edward M. Wu [1]. This failure criterion is based on the strength of anisotropic materials, making it applicable to composite laminates. Furthermore, Tsai–Wu considers the total strain energy to predict failure and can be used to determine the safety factor of orthotropic shells. A detailed study and application of the Tsai–Wu failure criterion were provided in the research [25,26] and expressed as a quadratic form for in-plane stress in a 2D state (*τ_23_* = 0, *τ_13_* = 0, *σ_3_* = 0). The general form of this criterion is given in Equation (2):

(2) $$F\left(\sigma_{k}\right)=F_{i}\sigma_{i}+F_{ij}\sigma_{i}\sigma_{j}$$

where j=1,2…6, $F_{i}$ and $F_{ij}$ are strength tensors, while $\sigma_{i}$ and $\sigma_{j}$ are stress tensor components. This equation is invariant, and each stress component reflects an independent material property. The independent nature of the failure criterion implies that the components do not interact with each other. Since the strength components are represented as tensors, this criterion allows their transformation into fiber directions for a specific ply. Equation (3) can be expressed as an inequality:

(3) $$F_{ii}F_{jj}-{F_{ij}}^{2}\geq 0$$

**Figure 6:**
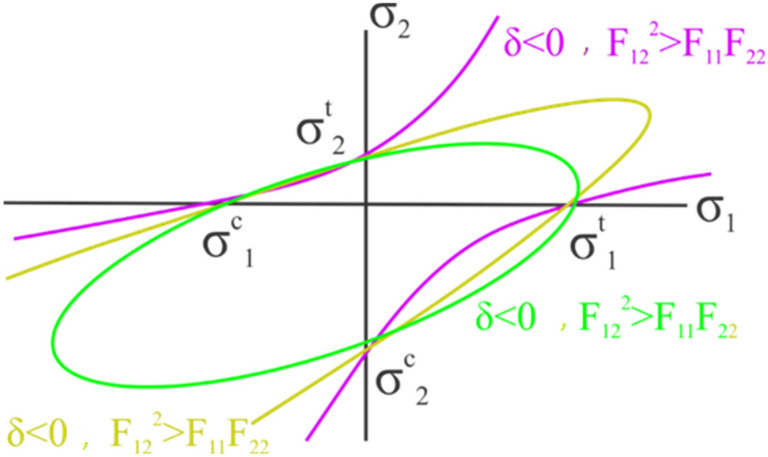
Three-dimensional failure envelope for the Tsai–Wu failure criterion [25].

Equation (3) indicates that repeated indices are not summations but individual diagonal members within the tensor matrix. Figure 6 illustrates the inequality (Equation (3)). This inequality ensures that failure will intersect each axis of the shear stress diagram without generating a hyperbolic function. For UD composite materials, a characteristic parameter (δ) is used for this type of material: ($\delta =4-\frac{\sigma_{2}^{t}\sigma_{2}^{c}}{\tau_{23}^{2}}$). When δ<0, as shown in Figure 6, it indicates that some laminate properties in the laminate do not meet the conditions required for a UD composite ply, and in such cases, some plies result in negative δ.

In addition to requiring that the strength tensor is positive, the criterion assumes tensor symmetry. This assumption reduces the number of independent members to six and twenty-one for $F_{i}$ and $F_{ij}$, respectively. By introducing another assumption, that the change in the sign of shear stress does not affect failure, the number of independent members is further reduced to three and nine for $F_{i}$ and $F_{ij}$, respectively. For composite materials, under the assumption of a plane stress state, the strength tensors can be written as

(4) $$F_{i}=\left(\begin{array}{c} F_{1}\\ F_{2}\\ F_{6} \end{array}\right)$$

(5) $$F_{ij}=\left(\left[\begin{array}{ccc} F_{11} & F_{12} & F_{16}\\ F_{12} & F_{22} & F_{26}\\ F_{16} & F_{26} & F_{66} \end{array}\right]\right)$$

Considering the nature of orthotropic materials, where $F_{6}=F_{16}=F_{26}=0$, the number of independent components reduces to six. If all these assumptions are applied to Equation (3), the simplified form of the Tsai–Wu failure criterion follows:

(6) $$I_{F}=F_{1}\sigma_{11}+F_{2}\sigma_{22}+F_{11}{\sigma_{11}}^{2}+F_{22}{\sigma_{22}}^{2}+F_{66}{\sigma_{12}}^{2}+2F_{12}\sigma_{11}\sigma_{22}\leq 1$$

where

(7) $$F_{11}=1/\sigma_{1}^{t}\sigma_{1}^{c}$$

(8) $$F_{1}=1/\sigma_{1}^{t}-1/\sigma_{1}^{c}$$

(9) $$F_{22}=1/\sigma_{2}^{t}\sigma_{2}^{c}$$

(10) $$F_{2}=1/\sigma_{2}^{t}-1/\sigma_{2}^{c}$$

(11) $$F_{12}=-\frac{1}{2}\sqrt{F_{11}F_{22}}$$

(12) $$F_{66}=1/{\left({\tau_{12}}^{F}\right)}^{2}=1/\sigma_{12}^{t}\sigma_{12}^{c}$$

Substituting these expressions into $\partial \sigma_{y}/\partial y+\partial \tau_{yx}/\partial x+\partial \tau_{yz}/\partial z+Y=0$ if it is substituted into the force equilibrium equation along the direction of the laminate stacking, the following form is derived:

(13) $$\sigma_{1}^{2}/\sigma_{1}^{t}\sigma_{1}^{c}+\sigma_{2}^{2}/\sigma_{2}^{t}\sigma_{2}^{c}+{\tau_{12}}^{2}/{\left({\tau_{12}}^{F}\right)}^{2}+\sigma_{1}\left(1/\sigma_{1}^{t}-1/\sigma_{1}^{c}\right)+\sigma_{2}\left(1/\sigma_{2}^{t}-1/\sigma_{2}^{c}\right)+2F_{12}\sigma_{1}\sigma_{2}$$

where $F_{12}$ is a coefficient related to principal stresses $\sigma_{1}$ and $\sigma_{2}$ and can be determined experimentally through laminate testing in both principal axes. If data is unavailable, $F_{12}$ can be estimated using the following expression:

(14) $$F_{12}\approx -\frac{1}{2}\sqrt{F_{11}F_{22}}=-1/2\cdot 1/\sqrt{\sigma_{1}^{t}\sigma_{1}^{c}\sigma_{2}^{t}\sigma_{2}^{c}}$$

Using material properties, specifically tensile strength components for desired directions, the components in Equation (6) can be expressed as

(15) $$F_{1}=1/X_{t}+1/X_{c}\;F_{2}=1/Y_{t}+1/Y_{c}\;F_{11}=-1/X_{t}X_{c}\;F_{22}=1/Y_{t}Y_{c}\;F_{66}=1/S^{2}$$

Here, $X_{t}$ and $X_{c}$ are the tensile and compressive strengths in one force direction, while $Y_{t}$ and $Y_{c}$ are the same characteristics in the perpendicular direction. These values can be found in the available literature or determined experimentally by testing specimens with the desired stacking sequence. By substituting the derived expressions into Equation (6), $F_{12}\;$ can be expressed as

(16) $$F_{12}=1/2\sigma_{biax}^{2}\left[-\left(1/X_{t}+1/X_{c}+1/Y_{t}+1/Y_{c}\right)\sigma_{biax}+\left(1/X_{t}X_{c}+1/Y_{t}Y_{c}\right)\sigma_{biax}^{2}\right]$$

or

(17) $$F_{12}=f^{*}\sqrt{F_{11}F_{22}}$$

where $\sigma_{biax}$ is the equivalent stress for both directions at failure, and 1≥f∗≤1. If material properties are known, the equivalent stress in both directions improves failure analysis accuracy; otherwise, Equation (17) is used.

**Figure 7:**
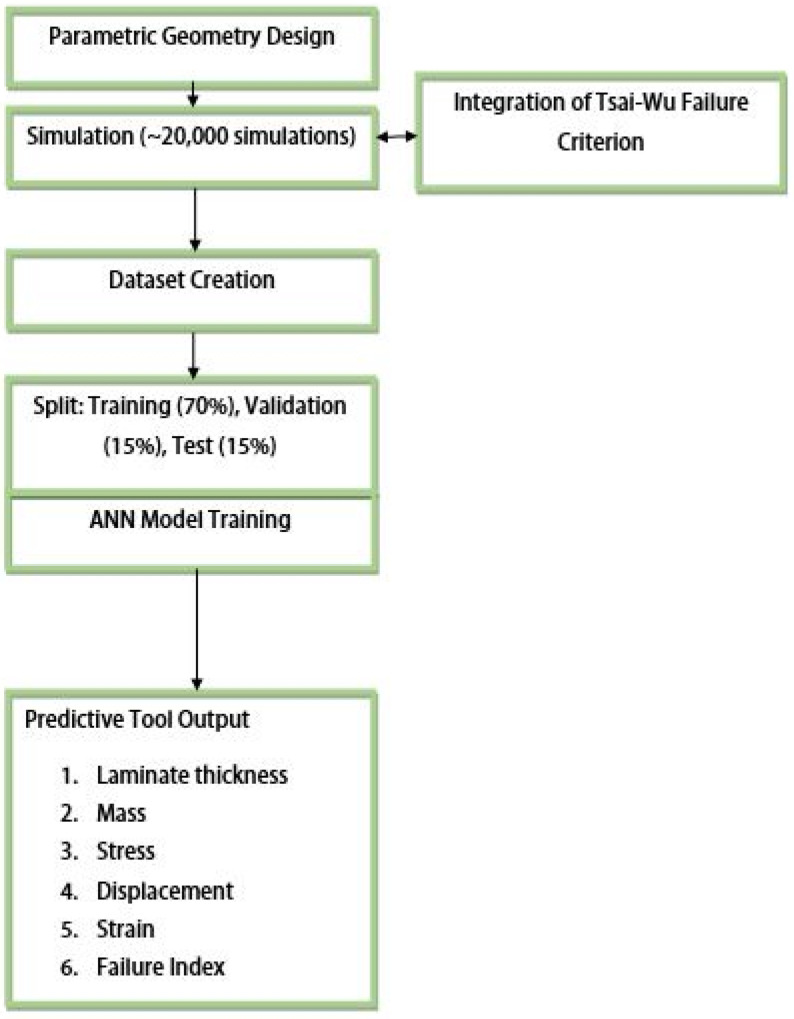
Illustration of the algorithm for developing a machine learning model for a predictive tool.

An illustration of the algorithm for developing a machine learning model for a predictive tool is shown in Figure 7. The diagram presents the key steps: parametric geometry design, database generation through high-fidelity simulations, training of artificial neural networks, and integration of the Tsai–Wu failure criterion. The resulting model enables the rapid and accurate prediction of structural performance based on input parameters.

## 4. Machine Learning Model

The created ANN model is used to predict six target variables: total thickness, mass, maximal displacement, stress, strain, and the structure’s failure factor coefficient, based on 14 input variables (layer thickness *t_i_* and orientations *θ_i_*, respectively, and hole diameters *R*_1_ and *R*_2_). Input parameters are quantities that determine the nature of the output results. The thickness and orientation of the layers define the structure and affect the types of loads it can withstand. In addition, the layer thicknesses define the total mass of the structure, while the hole diameters *R*_1_ and *R*_2_ determine the geometric characteristics of the structure based on the specified type of loading.

The ANN model is set to be a feedforward neural network (FFNN). This neural network represents a multilayer perceptron (MLP), where data propagates through multiple layers of neurons, including input layers, hidden layers, and the output layer. The hidden layers consist of multiple neurons that process the input data before passing it to the subsequent layer. Regarding the transfer function, an appropriate activation function is automatically selected for each layer of the neural network. These are typically sigmoid functions, hyperbolic tangent sigmoid functions, or linear functions. The type of network employed is a feedforward backpropagation network. Backpropagation occurs from the output layer to the input layer, calculating the gradient of the error function for each weight coefficient using the chain rule. Unlike the original direct approach of calculating the gradient for each layer separately, this backward flow of error information allows for efficient gradient computation one layer at a time. The transfer functions calculate the layer’s output based on its net input. This function receives an input matrix M with dimensions S × Q (where S is the number of neurons in the layer and Q is the number of variables), representing the network’s input vectors. The transfer function transforms each element of matrix M so that the output falls within the range of −1 to 1 [27].

The training function of the network is based on the Levenberg–Marquardt (LM) algorithm due to its stability and faster convergence. This algorithm provides numerical solutions by minimizing nonlinear functions. The LM algorithm interpolates between the Gauss–Newton method and steepest descent algorithms, combining the speed of the former with the stability of the latter. The primary concept of the LM algorithm is to execute a combined training process, where the steepest descent algorithm is used in regions with complex curvature until the curvature stabilizes sufficiently for the quadratic approximation (Gauss–Newton algorithm) to be performed [27,28]. FFNN can have an arbitrary number of layers and neurons per layer, depending on the complexity of the problem being solved. No strictly defined formula determines the optimal network architecture; instead, its structure is most often determined experimentally, based on experience, previous studies, or optimization procedures, such as hyperparameter search or evolutionary algorithms.

**Figure 8:**
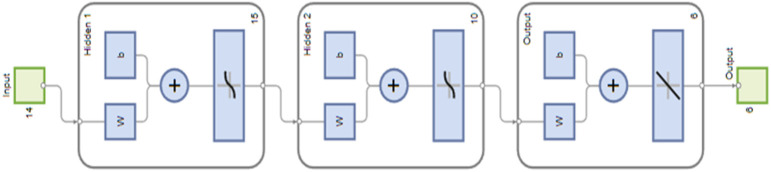
The neural network model formation.

The model uses 14 input parameters processed through a multilayer neural network with two hidden layers, resulting in a significant number of coefficients and high computational complexity. Consequently, the training and evaluation process of the model is computationally demanding, especially since most solutions are processed through multiple iterations. Precisely for this reason, it is necessary to choose the network architecture well, so that it is sufficient to make adequate predictions, but also simple enough so that its training is not too time-consuming. The final neural network architecture, determined empirically through iterative testing and performance evaluation, consists of two hidden layers: the first hidden layer contains 15 neurons, while the second contains 10 neurons. This neural network architecture allows the network to learn complex relationships between input and output variables. The dataset is divided into training, validation, and testing sets in proportions of 70%, 15%, and 15%, respectively, ensuring an adequate evaluation of performance. In addition to this split, the networks were tested with various combinations of dataset partitions. Finally, the results are computed through a single output neuron for each target variable. The flowchart of the neural network model formation is shown in Figure 8.

### 4.1. Neural Network Performance

To effectively model the complex relationships present in the dataset, the neural network model was used to establish correlations between input and output variables, where the outputs represent both the targets and constraints. This model is capable of capturing nonlinear dependencies between multiple input variables and their corresponding outputs, making it well-suited for the problem at hand.

**Figure 9:**
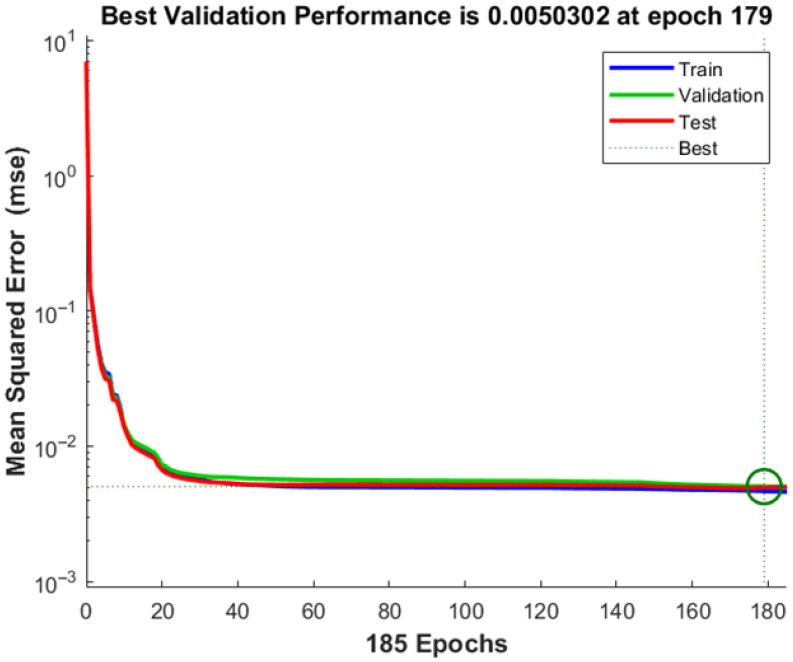
The mean-squared error of the neural network model (green circle is point with Best Validation Performance).

One of the key parameters for assessing the quality of the neural network is the mean-squared error during the model training process. Figure 9 shows the diagram of the mean-squared error variation through epochs.

The diagram indicates that the error decreases as the number of epochs increases. The decrease in mean-squared error over the training epochs indicates the model’s progressive learning and improvement in prediction accuracy. Monitoring this metric allows for early stopping to prevent overfitting and ensures that the trained network generalizes well to unseen data. Lower mean-squared error values suggest high predictive capabilities of the neural network, demonstrating its efficiency in linking input variables to their corresponding outputs.

The best value of mean-squared error 0.0050302, was achieved after 185 epochs.

**Figure 10:**
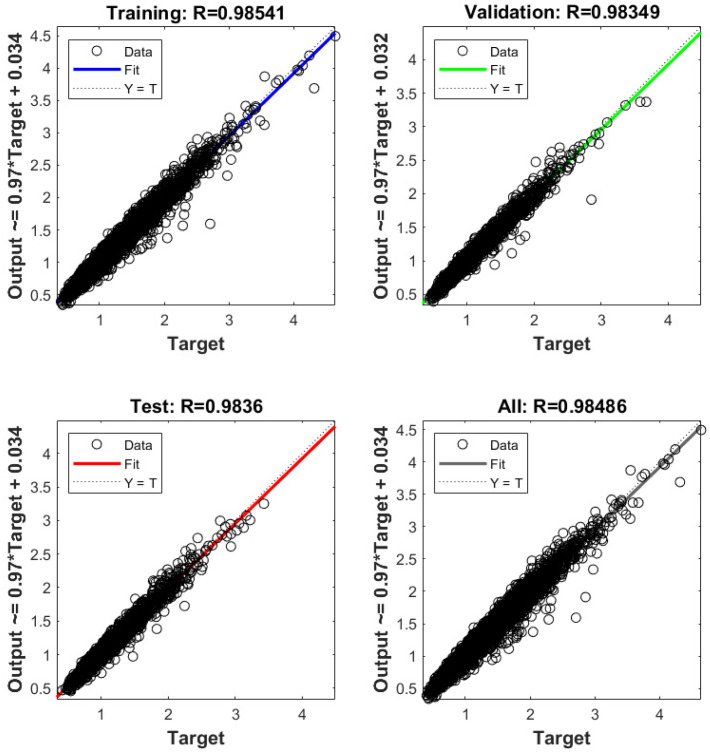
The relationship between actual and target values, predicted by the neural networks.

In addition to evaluating the mean-squared error, the model’s regression was also considered. The regression of the artificial neural network model is presented in Figure 10. The coefficient of determination, R^2^, was used to evaluate the model, where its squared values tend towards 1, indicating a strong correlation between the network’s predictions and the input data obtained from repeated FEA calculations. The solid line indicates the best linear regression between the output and target values. The complexity of the model also affects its predictive performance, but both the mean-squared error and the coefficient of determination confirm the high accuracy of the proposed model.

**Figure 11:**
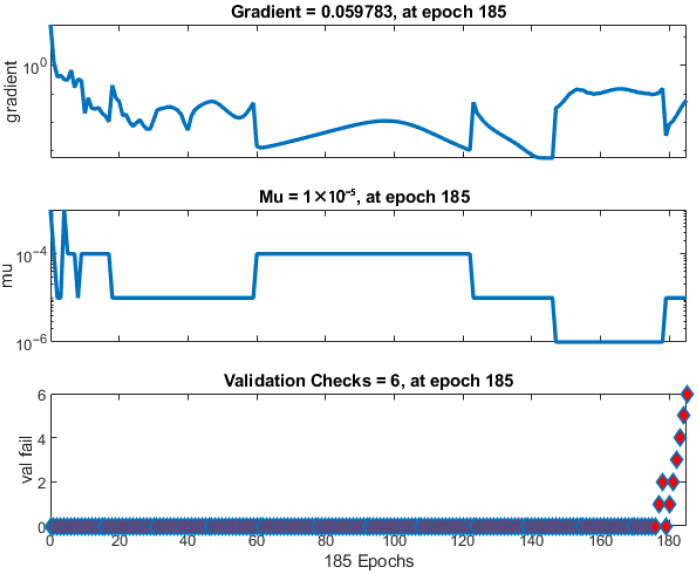
The training process flow.

The gradient size, momentum updates (Mu), and the number of validation checks per epoch were used to track the progress of the neural network training process (Figure 11). Mu is an adjustment parameter used in the optimization process to avoid local minima. The diagram for Mu versus epochs shows the model’s stabilization during training. The gradient fluctuates during learning, but as the number of epochs increases, the losses decrease.

The datasets for training, testing, and validation show a strong correlation, with the test values aligning with those in the existing literature [28,29].

**Figure 12:**
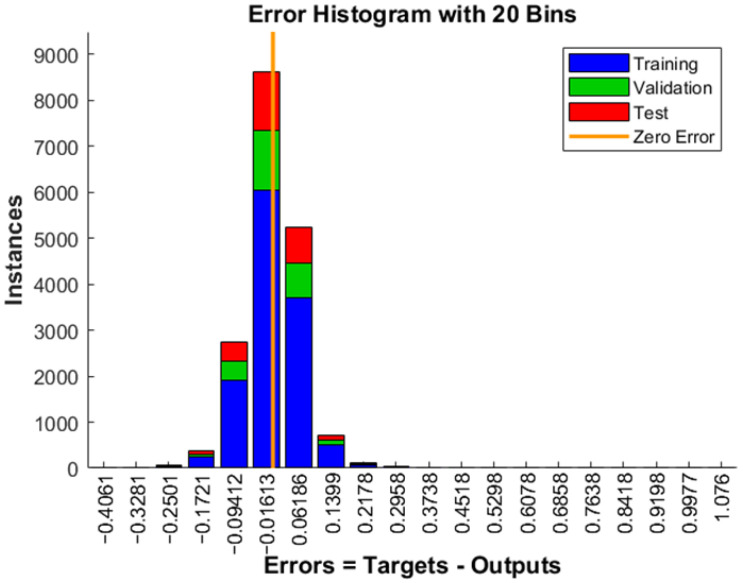
Error diagram of the process.

Early stopping is a technique used to avoid overfitting the machine learning model. During training, the model’s performance is regularly checked on the validation set. If performance does not improve over a certain number of epochs, training is stopped earlier. The diagram showing validation indicates that training will stop if the validation metric does not improve over the last six epochs. If performance does not improve during these epochs, it suggests that the model has reached its optimal performance and further training may lead to overfitting. The error between the target values and the predicted values after training the artificial neural network for one output (composed of six independent networks) is shown in Figure 12. Basic overfitting mitigation techniques that were applied, in addition to early stopping, were simplification of the starting parameterized model (that included only the most important geometric and material features), sufficient size of the training data (that were formed in such a way to ensure sufficient variability), regularization by limiting large weights, and cross-validation by testing different data splits. Also, ANN generation and training processes were repeated several times to ensure repeatability.

The error values indicate the degree to which the predicted values differ from the desired target values. The vertical axis (instance) represents the number of data samples, with the zero-error line corresponding to the zero-error value on the *x*-axis. The zero-error line on the diagram is around the value of −0.01613. There is a strong correlation between the predictions and the target, confirming that the preprocessing steps on the dataset, using the selected training model, were well-suited to the problem addressed in this paper.

### 4.2. Failure Prediction Tool

The developed tool for predicting the characteristics of composite materials uses advanced machine learning techniques to quickly and accurately assess the key parameters of structural behavior. The tool was developed based on a large database generated through repeated FE simulations under various loading conditions and combinations of material layers. This tool enables the prediction of parameters such as stresses, displacements, deformations, mass of the structure, and safety factors based on the input of geometric and structural parameters. Using this predictive tool, the performance of composite structures can be quickly assessed under different scenarios, contributing to a more efficient design and optimization process. The developed tool, based on randomly selected input data, provides predictions for six desired results.

**Figure 13:**
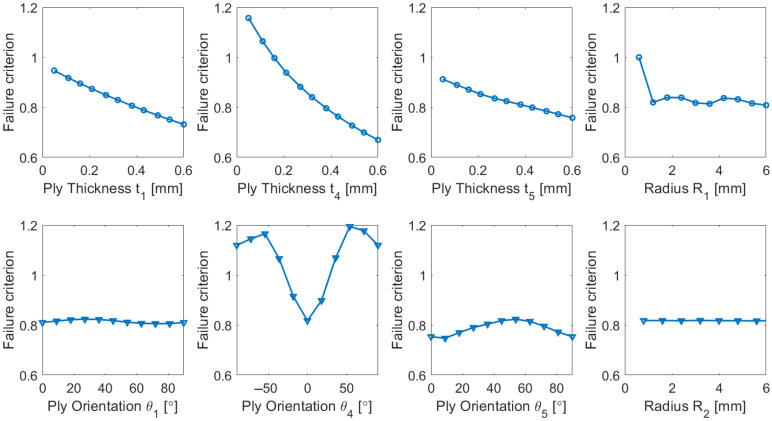
Sensitivity analysis of the representative input parameters.

When aerospace structures of complex geometry are involved, the output may depend on numerous input parameters, and their individual effects should be ascertained. Similarly to [30], in the current study, a simple sensitivity analysis was also performed. A mean solution (with the value of each input parameter in the middle of the design space) was initially chosen, and then each parameter was independently varied within the allowed boundaries, and its effect on one of the outputs was estimated. Figure 13 illustrates the sensitivity of several inputs to the failure criterion. For solutions in the inner part of the inspected design space, it can be observed that all input parameters affect structural reliability, particularly ply thicknesses. As expected, ply orientation is very significant for unidirectional plies (here, in layer 4), and somewhat less meaningful for bidirectional plies (here, applied in layers 1–3, 5, and 6). The inspected hole radii appear less influential to failure criteria but affect maximal deformations more significantly. Therefore, for obtaining a satisfactory solution, one should consider the complete set of inputs and outputs, and a more detailed sensitivity analyses (applicable near the boundaries of the design space) will be performed in the future.

**Figure 14:**
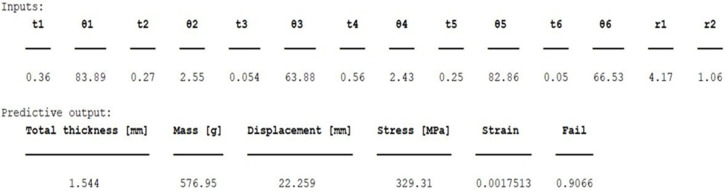
The output of the predictive tool.

Each conducted case results in a prediction for selected input parameters. The input parameters chosen for testing the predictive tool are also utilized in the FEA to validate its accuracy. The output generated by the tool, following the input of the selected parameters, is presented in Figure 14.

## 5. Results and Discussion

To further illustrate and validate the machine learning model, an additional static analysis using the Finite Element Analysis (FEA) was conducted on an arbitrarily chosen model, and the results were compared with the predictions from the ML model. The developed parameterized beam model and the boundary conditions used in the analysis served as the basis for generating the dataset used to train the artificial neural network (ANN). The input data varied in terms of individual layer thicknesses, their orientations, hole diameters, and both the magnitude and nature of the applied load. The goal of the tool, based on all scenarios for which the model has been trained and validated, is to identify the corresponding case (based on the given input parameters) and provide a prediction accordingly.

**Figure 15:**
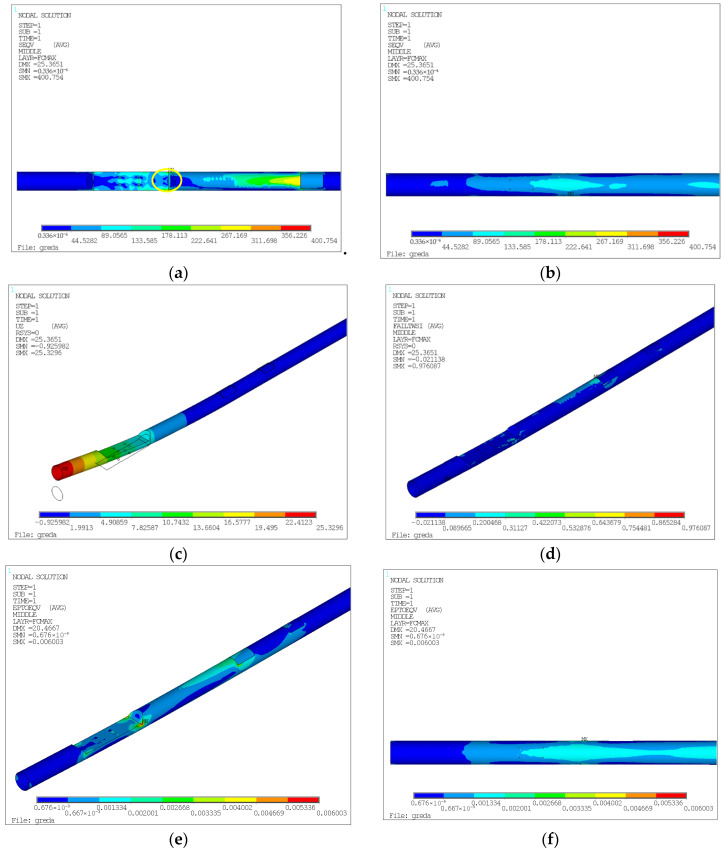
(**a**) Maximum stress for the entire laminate based on the failure criterion (top of the beam). (**b**) Bottom of the beam, with optimized hole sizes. (**c**) Beam displacement. (**d**) Failure factor coefficient for the entire beam. (**e**) Deformation of the beam (top). (**f**) Deformation of the beam (bottom).

For the model defined by the inputs given in Figure 14, and based on the integrated Tsai–Wu failure criterion, the predictive tool provides the values of maximum stress, displacement, and deformation for the entire laminate. Such values are comparable to the numerical model, as the maximum stress, displacement, and deformation can also be determined in FEA based on the same criterion. The results of the numerical simulation according to the Tsai–Wu criterion are shown in Figure 15.

In Figure 15, the singularity is marked with a circle, indicating the location of the maximum stress. This stress is due to a sharp contour and the insufficient fineness of the mesh. Nearby stresses are approximately 311.70 MPa. The maximum deformation value is also observed around these elements, with displacement outside the singularity considered to be about 0.20%. Additionally, the stress state at the bottom of the beam is more favorable, largely due to the reduction in the mounting hole from 8 mm to 1 mm.

**Figure 16:**
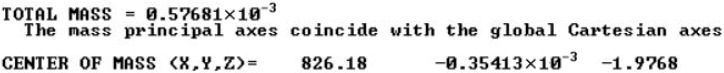
Beam mass for the case of layer arrangement, obtained through FEA.

Figure 16 shows the beam mass also obtained from the numerical model for the input parameters defined in Figure 14.

**Figure 17:**
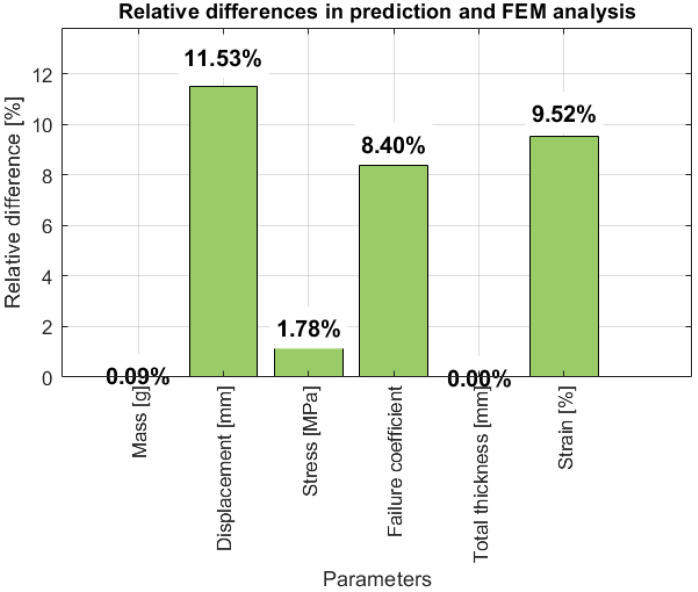
Relative differences between predicted values and FEA.

The relative differences between the output values obtained through prediction and the FE analysis are given in a table (Table 2) and also in a diagram (Figure 17).

The failure prediction tool was developed as a preprocessing step in the design of composite structures. Its limitations lie in its predictive capabilities, which are restricted to a single, unique geometry for which the model was trained. However, the model demonstrates highly satisfactory results for the structural element for which it was developed, with relative differences between predicted and actual values falling within acceptable limits. The relative differences for mass, stress, and total thickness remain below 2%, while those for failure coefficient, displacement, and deformation stay below 12%. This comparison validates the artificial neural network model, confirming its effectiveness in accurately predicting key structural parameters, thereby supporting its application in composite material design. Furthermore, this tool holds significant potential for integration into aircraft maintenance systems. With certain improvements and the development of models for each structural component of the aircraft, the process of monitoring the service life of structural elements can be substantially accelerated, while reducing the costs of testing by various methods. Although originally developed for a specific engineering application, the proposed predictive tool is highly modular, enabling its adaptation to a broad spectrum of biomedical domains. Potential applications include real-time prediction and control in assistive mobility systems, rehabilitation robotics, and patient-specific modeling, where integration with sensor networks and physiological data is essential for enhanced functionality and user-specific customization.

## 6. Conclusions

The results obtained from extensive numerical simulations (approximately 20,000 individual analyses) played a pivotal role in the construction of a comprehensive database, which served as the foundation for the development of an advanced machine learning model, specifically an artificial neural network (ANN). The developed ANN model demonstrated exceptional predictive accuracy, with the correlation between the predicted and numerically obtained values consistently remaining within a 15% margin. This level of agreement is considered highly satisfactory, confirming the robustness of the model in forecasting the key mechanical parameters of composite structures under various loading scenarios.

One of the primary advantages of this approach lies in its ability to reliably predict the mechanical behavior of composite structures by employing the Tsai–Wu failure criterion. This capability is particularly valuable during the early phases of design, where different configurations of composite materials are being evaluated. By enabling the prediction of critical parameters such as stress, displacement, deformation, and failure initiation, the model significantly accelerates the design process, allowing engineers to make informed decisions without the need for expensive and time-consuming physical experiments or conventional numerical analyses.

This predictive tool represents a substantial advancement in the field of structural design and analysis by dramatically reducing the time typically required for traditional Finite Element Analysis (FEA). In contrast to conventional approaches, which may require several days of complex simulations, the proposed model can deliver accurate results within minutes, thereby enhancing decision-making efficiency. Furthermore, once the database is established, there is no further need for additional FE simulations or geometric remodeling, which substantially improves the model’s flexibility and operational efficiency. Importantly, owing to its stochastic nature, the ANN model complements deterministic FE simulations, which are inherently limited in capturing complex or partially understood physical phenomena. The data-driven framework of the ANN allows for the incorporation of uncertainties and approximations of behaviors that are difficult to describe using conventional analytical formulations. In addition, its high computational efficiency and rapid response make the model a strong candidate for real-time applications, including inspection processes and the definition of predictive maintenance strategies in operational environments.

Looking ahead, numerous directions for further development and refinement are evident. Although the model has proven highly effective in predicting mechanical properties and failure points, expanding its applicability to a broader spectrum of structural configurations and loading conditions remains a priority. This may involve incorporating complex multiaxial load scenarios, variable environmental influences, and more sophisticated geometries, all of which would enhance the model’s utility in advanced engineering contexts. Moreover, integration with real-time data acquisition systems would enable dynamic model adaptation, allowing it to evolve in parallel with incoming data. The continued enhancement of this tool will be vital in addressing a wide array of structural optimization challenges—not only within the aerospace sector but also across automotive, civil, and biomedical engineering disciplines. There are already numerous examples of research in the development of contemporary biopolymers and biocompatible composite materials and structures [31,32,33,34,35]. The methodology presented here can also be applied to facilitate and accelerate their design, estimation of their structural features, and reliability analysis. The potential applications of this predictive model are vast, ranging from the optimization of lightweight aerospace structures to the development of more resilient, efficient, and cost-effective engineering solutions.

Ultimately, the ongoing development of this model represents a transformative step forward in the intersection of computational mechanics and machine learning, with the potential to revolutionize the methodology of structural analysis and failure prediction in the near future.

## Figures and Tables

**Table 1:** Mechanical properties of the materials.

Carbon Fiber Laminate					
Layer orientation		0°	90°	±45°	Epoxy resin Araldite LY3505
Young’s modulus (GPa)	*E*_11_	139.40	7.66	9.85	3.50
	*E*_22_	7.66	139.40	9.85	
	*E*_33_	7.66	7.66	7.66	
Shear modulus (GPa)	*G*_12_	3.680	3.680	7.070	1.296
	*G*_13_	3.680	2.940	2.940
	*G*_23_	2.940	3.680	2.940	
Poisson’s ratio	*ν*_12_	0.260	0.014	0.340	0.350
	*ν*_13_	0.260	0.304	0.304	
	*ν*_23_	0.304	0.260	0.304	
Tensile strength (MPa)		1500	50	110	85
Shear strength (MPa)		86			

**Table 2:** Comparison of machine learning model results (Figure 14) and FEA.

	Prediction Value	FEM Analysis Value	Relative Differences [%]
Mass [g]	577.32	576.81	0.09
Displacement [mm]	22.41	25.33	11.53
Stress [MPa]	317.24	311.70	1.78
Failure coefficient	0.894	0.976	8.40
Total thickness [mm]	1.54	1.54	0.00
Strain [%]	0.19	0.21	9.52

## Data Availability

The raw data supporting the conclusions of this article will be made available by the authors on request.
